# A case report of surgical repair of a post-catheterization radial pseudoaneurysm

**DOI:** 10.11604/pamj.2022.41.261.29725

**Published:** 2022-03-31

**Authors:** Spyros Ioannis Papadoulas, Polyzois Tsantrizos, Natasa Kouri, Andreas Tsimpoukis, Nikolaos Koutsogiannis, Konstantinos George Moulakakis, Stavros Konstantinos Kakkos, Periklis Davlouros

**Affiliations:** 1 Department of Vascular Surgery, University Hospital of Patras, Rio, Patras, Greece,; 2 Department of Cardiology, University Hospital of Patras, Rio, Patras, Greece

**Keywords:** Radial access, radial pseudoaneurysm, case report

## Abstract

We report an 83-year-old patient with a huge post-catheterization right radial pseudoaneurysm, presented 17 months after a coronary angiography. Cases of radial post-catheterization pseudoaneurysms with a similar size are scarce in the literature. Delay in presentation led to painful skin ischemia due to tension, a sign of imminent rupture, which is also rare in the literature. Symptomatology included severe wrist pain and clinical signs consisted of a pulsatile painful mass in the right distal forearm. Management consisted of surgical excision and ligation of the radial artery in an urgent base. This case emphasizes the need for early diagnosis and management of post-catheterization pseudoaneurysms as delay may lead to severe enlargement with skin necrosis and imminent rupture. Ligation of the radial artery is an acceptable option when reconstruction of the artery is troublesome, provided that the palmar arch remains patent.

## Introduction

Radial artery pseudoaneurysm (PSA) after radial access for coronary procedures is an exceptionally rare complication with an incidence of <0.1% and with possible unfavorable results, if left untreated [1]. Up to date, apart from case reports, only a few large series exist examining the incidence of complications after radial access for coronary angiography [1]. Additionally, there are no clinical trials regarding the best treatment of PSAs, and consequently no systemic guidelines exist [2-4]. We present a patient with a painful radial pseudoaneurysm, treated urgently with ligation and discuss alternative treatment options, as well as preventive measures.

## Patient and observation

An 83-year-old patient was admitted with chest pain. He was an ex-smoker and suffered from arterial hypertension and hyperlipidemia. He underwent coronary angiography via right radial artery access. After a single puncture, a 5-Fr introducer sheath was placed. Coronary angiogram revealed mild coronary disease which was treated conservatively. A hemoband was placed to accomplish hemostasis.

**Figure 1:**
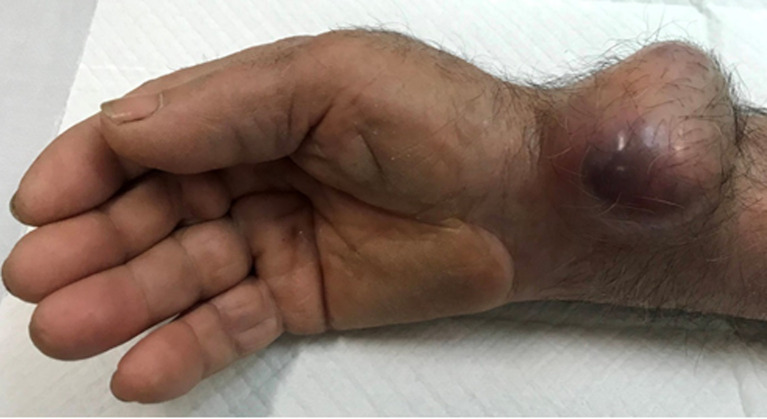
post-catheterization radial pseudoaneurysm at the external surface of the distal forearm measuring 6 x 5 cm in diameter; cyanotic appearance medially represents local ischemic changes due to long-standing severe skin tension (preoperative photo-oblique view)

**Figure 2:**
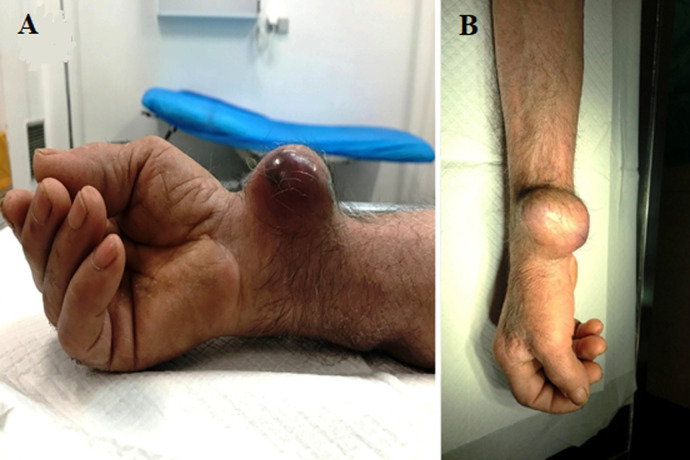
post-catheterization radial pseudoaneurysm: A) preoperative photo-frontal view; B) preoperative photo-side view

One week after discharge the patient noticed a small mass (around 1 cm in size) at the puncture site. This remained stable for a year and his physician suggested observation. However, the mass begun to enlarge during the next five months, and the patient presented to the emergency department due to severe pain in his right wrist. On clinical examination a remarkable mass was seen, pulsatile and tender, sizing 6 x 5 cm (Figure 1, Figure 2 A, B). Pain was radiating to the fingers. The Allen test was normal. The diagnosis of a radial artery post-catheterization PSA was set. Other possible diagnoses like a cyst, a lipoma, abscess, etc. were excluded due to the pulsatility of the mass and the relevant history of vascular puncture. Due to ischemic overlying skin and severe pain the patient was transferred to operating theatre, emergently. No colour duplex or computed angiography was performed.

**Figure 3:**
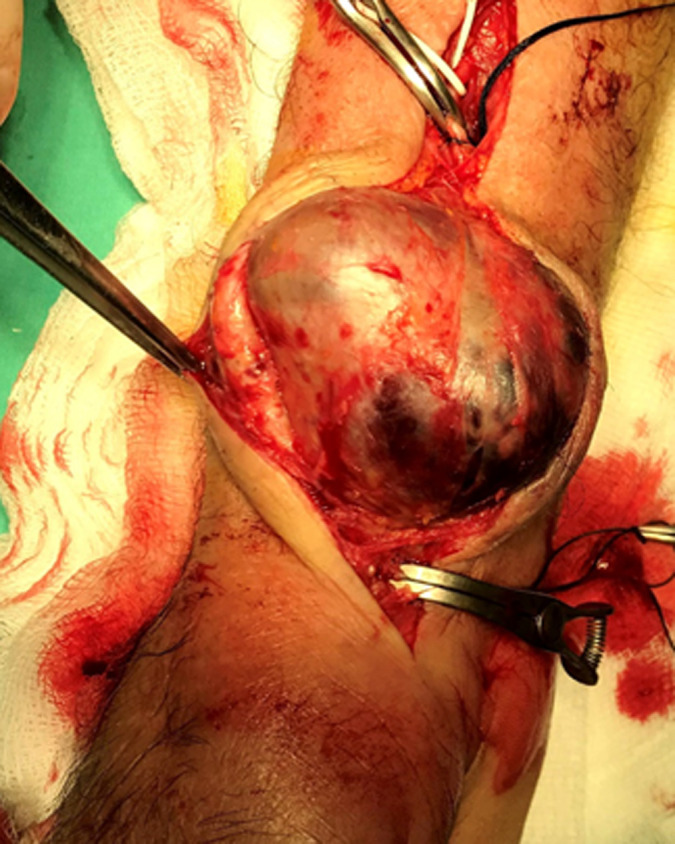
both the proximal and distal radial artery have been looped and clamped with bulldog clamps; the sac of the aneurysm is dissected free from the superimposed skin

**Figure 4:**
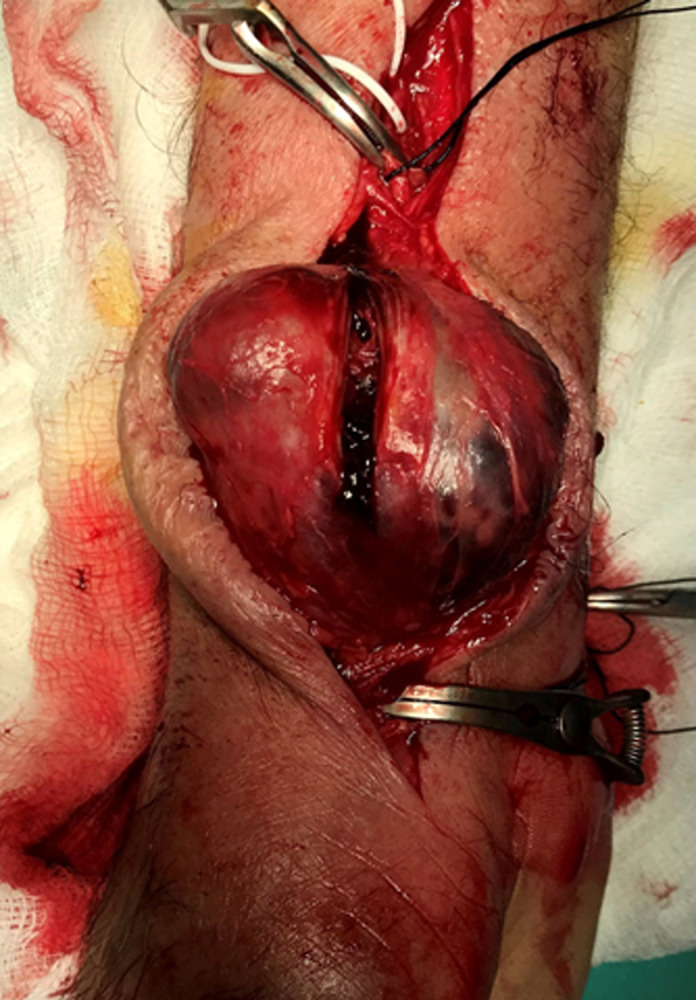
the fibrous wall of the sac (pseudo-capsule) is opened with a sharp blade; wall thrombus is seen through the incision

**Figure 5:**
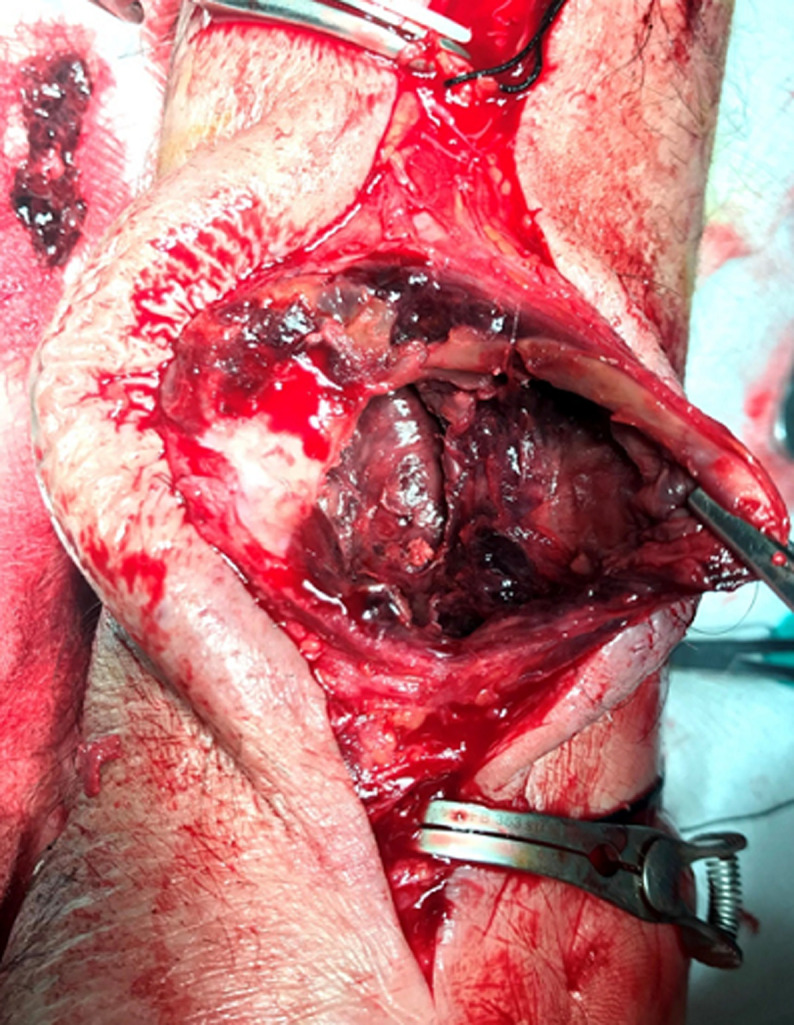
part of the sac is removed as well as the thrombus

**Figure 6:**
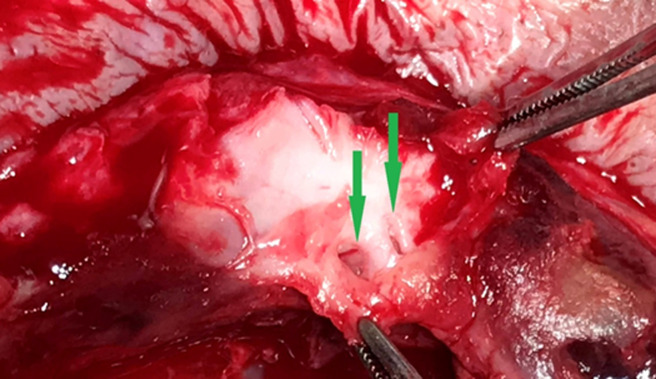
the lumen of the proximal and the distal radial artery is clearly seen (arrows); a partial transection of the radial artery from the sheath was presumably the causative process for aneurysm formation

Under local anaesthesia we removed the sac and evacuated the thrombus (Figure 3, Figure 4, Figure 5, Figure 6). We proceeded with ligation of the proximal and distal radial artery with silk suture No 0. We had this option as the Allen test was normal. An alternative procedure would include interposition of a vein graft between the two orifices. We decided against this, because of the strict adhesions with the nearby structures mainly nerves. The latter was due to the inflammatory reaction from the long-term tension to the local structures imposed by the aneurysm. Consequently, dissection of the arterial stumps was difficult and risky. His postoperative course was uneventful, and the patient discharged. Three months later the patient remains asymptomatic and satisfied with the medical care given.

## Discussion

Radial artery PSA develops from a tear through all the layers of the artery leading to formation of a contained hematoma, which eventually builds up a thin wall capsule, (false sac) from the surrounding tissues reaction [5]. There is inadequate thrombosis of the puncture hole after sheath retrieval [6]. It links with the feeding artery with a neck, whose width and length has implications for its treatment [2]. The PSA may appear in the puncture site or to a remote place centrally along the radial artery because of guide-wire injury. Radial artery pseudoaneurysms may present as a pulsatile mass, days, or weeks after the intervention. They might become painful as they grow up, rupture due to their wall frailty, or cause distal embolization [2]. Coexistent infection may lead to rupture and gross hemorrhage [7]. Most patients develop symptoms in the first week and do not need observation beyond 60 days [1].

Diagnosis is usually confirmed with color duplex. Specifically, the duplex depicts an anechoic mass with bidirectional internal flow (the “ying-yang” and “to-and fro” signs) [2,3]. Treatment aims to terminate the communication between the artery and the sac. It depends on the size, location, symptoms, infection, outflow and patency of the palmar arch. Small radial PSAs (diameter <3 cm) may thrombose spontaneously, in 4 weeks [2]. This also may happen in case of long narrow necks, and low flow volumes, in which case observation is suggested [2]. If they remain unchanged local compression can be applied [3]. This could be performed continuously with a compression bandage, a simple hemostatic device or a pneumatic one, over the PSA. It aims to restrict the inflow and to obliterate the neck without occluding the radial or ulnar flow [2]. A pulse oximeter may be placed at the fifth finger. Intermittent sessions may be used lasting from 20 minutes to 3 days. Our patient was not eligible for compression because the PSA was painful and tense with ischemic skin. Compression may be assisted with ultrasound (USGC treatment). The PSA is compressed with the probe aiming to eliminate the flow through the neck. Its effectiveness varies between 60-90% [2]. Infection, skin ischemia and frail appearance of the PSA are contraindications [2]. Some authors suggest occluding selectively the proximal radial artery (after identification with ultrasound) for 2-4 hours [4,7].

Radial artery pseudoaneurysm thrombosis can also be achieved with percutaneous thrombin injection under direct vision with ultrasound (USGTI) [3]. Special care is given to avoid dissemination of the thrombin in the radial artery through the neck. This technique is contraindicated in wide necks. It works faster achieving thrombosis in about 6 seconds, compared to about 45 minutes with USGC treatment [2]. It could cause PSA rupture. In our patient the severe pain prevented any compression procedures. There is one case report describing the placement of a covered-stent to exclude a radial PSA, which was introduced through the ulnar artery. Afterwards the PSA was evacuated with a syringe [8]. Embolization with ONYX^TM^ liquid embolic system (Medtronic, Irvine, CA) has been used to embolize a PSA at the anatomic snuff-box after right distal radial access [6]. Surgery should be considered if the previous strategies have not been effective (usually because of a large puncture hole). PSAs that remain active beyond 3 weeks, are larger than 3 cm or have been painful are eligible for surgery [9,10]. In our case all these parameters were present. Cases with a radial post-catheterization PSA´s with a similar size are scarce in the literature. The delayed presentation, 17 months after a coronary angiography, is also scarce in the literature. Open repair includes evacuation of the hematoma, excision of the sac and arterial repair [2]. We proceeded with ligation which is generally well tolerated since complete superficial and deep palmar arches are present in at least 80% and 90% of cases, respectively [10]. In our patient a partial transection of the radial artery from the sheath was presumably the causative process for PSA formation. Prevention of radial pseudoaneurysms include proper and meticulous use of guidewires and catheters, adequate wrist compression and early detection of a local hematoma [4]. These measures should be more intense in anticoagulated patients. In case of a hematoma the patient must be alert for the development of a pseudoaneurysm in order to inform promptly his physician [5].

## Conclusion

This case emphasizes the ligation of the radial artery as an alternative option to treat large symptomatic post-catheterization pseudoaneurysms. It is not advised as a rule but only in cases where artery reconstruction is troublesome and palmar arch remains patent. We should have in mind that skin necrosis is an impending sign of rupture and urgent treatment is imperative. Regarding prevention, the patients must be alert and inform the doctor should any swelling, ecchymosis or pain appears at the access site after the procedure for the next 2 months.
